# Catheter-related bacteremia due to Kocuria rosea in a patient undergoing peripheral blood stem cell transplantation

**DOI:** 10.1186/1471-2334-4-62

**Published:** 2004-12-22

**Authors:** Fevzi Altuntas, Orhan Yildiz, Bülent Eser, Kürsat Gündogan, Bulent Sumerkan, Mustafa Çetin

**Affiliations:** 1 Department of Hematology-Oncology and BMT Unit, Faculty of Medicine, Erciyes University, Kayseri, Turkey; 2 Clinical Microbiology and Infectious Disease, Faculty of Medicine, Erciyes University, Kayseri, Turkey; 3 Department of Microbiology, Erciyes University, Faculty of Medicine, Kayseri, Turkey

## Abstract

**Background:**

*Micrococcus species *may cause intracranial abscesses, meningitis, pneumonia, and septic arthritis in immunosuppressed or immunocompetent hosts. In addition, strains identified as *Micrococcus spp*. have been reported recently in infections associated with indwelling intravenous lines, continuous ambulatory peritoneal dialysis fluids, ventricular shunts and prosthetic valves.

**Case presentation:**

We report on the first case of a catheter-related bacteremia caused by *Kocuria rosea*, a gram-positive microorganism belonging to the family *Micrococcaceae*, in a 39-year-old man undergoing peripheral blood stem cell transplantation due to relapsed Hodgkin disease. This uncommon pathogen may cause opportunistic infections in immunocompromised patients.

**Conclusions:**

This report presents a case of *Kocuria rosea *catheter related bacteremia after stem cell transplantation successfully treated with vancomycin and by catheter removal.

## Background

Long-term indwelling central venous access devices are indispensable to the supportive care of cancer patients. Infection remains a major complication of Hickman type catheters [1]. Intravascular catheter-related infections can be the cause of morbidity and mortality in patients undergoing autologous peripheral blood stem cell transplantation (PBSCT). *Staphylococcus aureus*, coagulase-negative staphylococci, aerobic gram-negative bacilli and *Candida albicans *are the most frequent causes of catheter related bloodstream infection [2]. *Kocuria rosea*, is gram-positive, strictly aerobic microorganisms belonging to the family *Micrococcaceae *that usually grow on simple media and generally considered as non-pathogenic commensals that colonize the oropharynx, skin and mucosa. However, it can be opportunistic pathogen in the immunocompromised patient [3,4].

We describe the first case of central venous catheter (CVC) related infection associated with *Kocuria rosea *in a patient undergoing autologous PBSCT. He had a chills, high fever, tachycardia and increased respiratory rate. Persistent bacteremia, which was unresponsive to vancomycin, was successfully treated by catheter removal.

## Case presentation

A 39-year-old man with relapsed Hodgkin disease was admitted to Erciyes University Hospital for autologous PBSCT. A Hickman-type central venous catheter was implanted and the patient received a conditioning regimen consisting ifosfamide, carboplatin and etoposide. On day 3 after PBSCT, he was acutely ill with a chills, high fever (temperature 38.5°C), tachycardia (heart rate 108 beats/min) and increased respiratory rate (26 breaths/min). Clinical, radiological and microbiological diagnostic procedures were performed, and at three blood cultures were obtained from peripheral veins and catheter half an hour apart. The patient was empirically treated with intravenous imipenem/cilastatin, 2 g/day; and amikacin, 1 g/day. On day six after PBSCT, he had a persistent fever and the temperature spiked to 39.5°C. There was no sign of a catheter exit-site infection. Chest radiography was normal. Serial blood cultures taken from the catheter (two cultures) and a peripheral vein (four cultures) were positive for *Micrococcus spp*., which were identified as *Kocuria rosea*. An antibiogram showed that the pathogen was sensitive to ampicillin/sulbactam, erythromycin and vancomycin. Imipenem and amikacin were discontinued, and intravenous vancomycin, 2 g/day was begun. The patient did not respond satisfactorily to vancomycin therapy for five consecutive days. The catheter was removed and a catheter tip culture was obtained. The cultures demonstrated a massive growth of *Kocuria rosea*. Blood cultures were thereafter negative. The patient was discharged from the hospital after 14 days of intravenous vancomycin. He was followed for six months; the patient is alive and well and is in complete remission of the primary disease.

## Microbiological diagnosis

Cultures of blood from the peripheral veins and the CVC were performed with a BACTEC system (BACTEC 9210; Becton Dickinson). The CVC tip was cultured by using the Maki roll technique. A growth of more than 15 cfu on the blood agar plate was considered positive. It was identified as *Kocuria rosea *on the basis of Bergey's Manual of Systematic Bacteriology [5]. This identification was confirmed with the commercially available tests (ID32 Staph ATB system, and mini-API, Biomerieux).

## Discussion

*Micrococcus spp*. are usually regarded as non-pathogenic skin commensals and their clinical relevance is questionable. However, *Micrococcus spp*. can be opportunistic pathogens in immunocompromised patients such as PBSCT. It has been reported that *Micrococcus luteus *has been implicated as the causative agent in cases of intracranial abscesses, meningitis, pneumonia and septic arthritis in immunosuppressed or immunocompetent hosts [6-12]. In addition, strains identified as *Micrococcus spp*. have been reported recently in infections associated with indwelling intravenous lines, continuous ambulatory peritoneal dialysis fluids, ventricular shunts and prosthetic valves [13-18].

We performed a Medline search using the terms "Micrococcus" and/or "*Kocuria rosea*" and/or "Kocuria". However, we were unable to find published studies on catheter infections caused by *Kocuria rosea *in patients undergoing HSCT in febrile neutropenic period.

In this patient, the repeated isolation of *Kocuria rosea *from different blood cultures in the absence of other microorganisms, together with isolation of the same organism from the catheter were primarily responsible for the episodes of febrile neutropenia. The catheter was then removed and cultures demonstrated a massive growth of *Kocuria rosea*.

The clinician should not underestimate the importance of the repeated isolation of a *Kocuria rosea *from blood cultures. Although uncommon, the possibility that a central venous line may be the portal of entry should be evaluated. Adherence of bacteria to the silastic tube would possibly explain the failure of treatment by antibiotics alone.

## Conclusions

This report emphasizes that *Kocuria rosea *should be considered as a nosocomial pathogen, which may cause of catheter infections in febrile neutropenic patients. In these patients, persistent *Kocuria rosea *bacteremia unresponsive to medical management should be treated by catheter removal.

## Competing interests

The author(s) declare that they have no competing interests.

## Authors' contributions

FA, OY and KG carried out the clinical study of the patient. BS carried out the culture and specific identification of the bacterium. BE and MC drafted the manuscript. All authors read and approved the final manuscript.

## Pre-publication history

The pre-publication history for this paper can be accessed here:


